# Understanding intergenerational dynamics and social support’s impact on health and well-being of older adults in South Asia: a scoping review

**DOI:** 10.1186/s13643-025-02833-z

**Published:** 2025-04-11

**Authors:** Selim Jahangir, Divya Sussana Patil, Jagriti Gangopadhyay, Tobias C. Vogt

**Affiliations:** 1 Health Demography & Department of Social and Health Innovation, Prasanna School of Public Health, Manipal Academy of Higher Education, Manipal, India; 2 Centre for Evidence-Informed Decision-Making, Prasanna School of Public Health, Manipal Academy of Higher Education, Manipal, 576104 India; 3 Manipal Institute of Social Sciences Humanities and Arts, Manipal Centre for Humanities, Manipal Academy of Higher Education, Manipal, 576104 India; 4 Population Research Centre, Faculty of Spatial Sciences, University of Groningen, Groningen, The Netherlands; 5 Health Demography, Prasanna School of Public Health, Manipal Academy of Higher Education, Manipal, India

**Keywords:** Intergenerational relations, Ageing, Older adults, Migration, Well-being

## Abstract

**Background:**

Traditionally, family members take care of older relatives in South Asian societies, and there is a strong reciprocal filial obligation through intergenerational family relations. The changing family structure, living arrangements, and out-migration have led to subsequent changes in reciprocal family support that influence the health and well-being of older adults. This scoping review aims to (1) map the evidence and prevailing motivations for family support including financial, instrumental, and emotional support that affect the health and well-being of older adults and (2) identify the research gaps in the academic scholarship available on motivation for family support to older adults given the changing demographic and societal dynamics in the South Asian societies.

**Methods:**

We followed the Preferred Reporting Items for Systematic reviews and Meta-Analyses extension for Scoping Review (PRISMA-ScR) guidelines. Electronic databases PubMed, Embase, Scopus, and ProQuest were searched, and Google Scholar was used to identify grey literature. The screening of titles, abstracts, and full texts included 22 studies for analysis.

**Results:**

The included studies covered health indicators such as stress and/or depression, loneliness/isolation, loss of support/neglect, and level of satisfaction to illustrate well-being of older adults. The findings revealed that adult children recognised filial duties and responsibilities to provide care to older parents, whereas older parents provide cultural upbringing and care to grandchildren. Reciprocal care exchange, cultural expectations, and intergenerational transfers motivated adult children to be primary caregivers to their older parents. Intergenerational family care such as financial, instrumental, and emotional support is associated with a higher level of life satisfaction and lower level of depression and thus reported better health and well-being among older adults.

**Conclusion:**

Although intergenerational support is still a significant factor in determining the well-being of older adults in South Asia, this study shows the complexity of intergenerational ambivalence, where caregiving responsibilities lead to both emotional stress and a sense of obligation. Additionally, out-migration of adult children and subsequent physical absence also increase psychological distress and loneliness of older adults. This emphasises the need for policies that address both the emotional and financial aspects of elder care. The Madrid International Plan of Action on Ageing (MIPAA) policies can be adopted to ensure friendly and supportive environments and emphasise the health and well-being of older adults in developing countries. MIPAA highlights the importance of policies that promote intergenerational solidarity, active ageing, and social protection for older individuals.

**Supplementary Information:**

The online version contains supplementary material available at 10.1186/s13643-025-02833-z.

## Introduction

Social support is a key dimension of intergenerational family relationships, influenced by a variety of factors including economic circumstances, social norms, public policy development, longer lifespans, and longer dependency periods [1, 2]. Several previous studies in the Western context have documented that intergenerational family support influences the health and well-being of older adults due to co-residence and contact between generations [3]. The prevalence of intergenerational family support varies across cultures [4], and there is limited understanding of the issues that endorse and motivate family members to provide informal care in South Asian societies [5]. Herrera and colleagues mentioned that intergenerational relationships are strongly associated with the subjective well-being of older adults in terms of the quality of relationships, recreational activities, and family identity [6].

In addition, reciprocity in intergenerational support is important for the quality of life of older people [7]. Reciprocity strengthens emotional bonding and solidarity in the family which is a central element for the well-being of older adults [8]. In contrast, several studies reported intergenerational ambivalence as an alternative to solidarity especially in situations of family support [9]. Intergenerational ambivalence is the existence of conflicting emotions towards a parent or child [10]. Intergenerational ambivalence acknowledges the existence of conflicting emotions that individuals may experience towards their parents or children. It recognises that familial relationships are not always characterised by unyielding love and harmony but are often marked by a mixture of positive and negative feelings. This concept underscores the idea that individuals can simultaneously hold deep affection and appreciation for their family members while also grappling with frustration, resentment, or disappointment [3]. In developing societies like in South Asia, intergenerational ambivalence is arising from large-scale rural out-migration of young adults leaving children and older parents behind [11]. Likewise, increasing female labour force participation, changing family structure, and living arrangements have also resulted in challenges in caring for children and older parents [5]. Changing preferences and norms among younger generations have the potential to exacerbate intergenerational conflicts within families and societies. As societal values evolve, the discrepancy in beliefs, attitudes, and lifestyle choices leads to tensions and misunderstandings between age groups, thereby creating intergenerational conflicts [12]. Moreover, the ageing of societies in South Asia further accentuates the need for intergenerational care [13]. As the region experiences demographic shifts with a growing proportion of older populations, the demand for support, assistance, and healthcare services increases [14]. This situation places an additional burden on younger generations who must shoulder the responsibility of providing care and support to their ageing parents and relatives [15]. Moreover, family support becomes especially relevant in South Asian societies because of the lack of public old-age support systems [16].

Since the early 2000 s, studies have shown that the traditional informal support system is slowly weakening due to urbanisation and modernization and is not able to fulfil even the basic needs of older parents in South Asia [17, 18]. Several ethnographic and qualitative studies undertaken across Asia established that the widening generation gap, due to changing attitudes towards older adults and resultant tensions and conflicts, has led to weakening “filial piety” and subsequent reduction in respect [19–23].

In South Asian societies, reciprocal care exchange, cultural expectations, and intergenerational transfers motivate adult children to be primary caregivers to their older parents [24–26]. However, the changing family structure and the out-migration of adult children have also influenced ‘left behind’ older adults’ cultural expectations of care and hurt their health and well-being [11, 27]. In addition, imbalances in intergenerational family relations lead to renegotiation of their living arrangements, stress, loneliness, isolation, loss of basic support, and a sense of being neglected [28, 29]. Hence, for several reasons, it is important to map intergenerational support to older adults in South Asia. Firstly, it sheds light on the changing roles and expectations within families. As younger generations migrate for better opportunities, the responsibility of caring for ageing parents falls on a smaller pool of family members or even solely on the older adults themselves. Secondly, understanding intergenerational support is essential for promoting solidarity and social cohesion within communities. Finally, it will inform targeted policies, promote social cohesion, and drive innovative solutions to ensure good health and well-being.

Thus, this scoping review aims to focus on the research gaps of effects of long-term intergenerational support to older adults, emotional challenges of caregivers, and the implications of migration on older parents in South Asia. In addition, there is limited focus on formal support system and gender disparities in caregiving responsibilities. This study examines the impact of intergenerational support on the health and well-being of older people in South Asia in light of changing family dynamics and migration trends, thus providing a novel perspective on the subject. In this context, the scoping review aims to address the following research questions: (a) What are the cultural, emotional, economic, and social arrangements that motivate intergenerational support in South Asian societies and (b) what are the demographic and cultural dynamics that influence the health and well-being of older adults.

The following definitions are used in the scoping review:

1. An older person is defined as a person who is over 60 years of age [30]. It is also subject to the constructions by which each society makes sense of old age, and in many developing countries including South Asia, old age is seen at the point when active contribution is no longer possible [31].
2. Health is defined as “a state of complete physical, mental and social well-being and not merely the absence of disease or infirmity” [32]. Social determinants of health are “the conditions in which people are born, grow, work, live, and age, and the wider set of forces and systems shaping the conditions of daily life” [32].
3. Intergenerational support covers the different forms of assistance between parents and children, including financial, instrumental, and emotional support [33]. In this scoping review, we considered intergenerational family support as any family-based informal support given by young adult children and grandchildren or vice versa.

## Material and methods

The proposed scoping review was conducted following the Joanna Briggs Institute (JBI) methodology for scoping reviews. The review adopted Preferred Reporting Items for Systematic reviews and Meta-Analyses extension for Scoping Reviews (PRISMA-ScR) guidelines [34] and followed the five-stage methodological protocol of identifying the research question, identifying relevant studies, study selection, charting the data, and collating, summarising, and reporting results as outlined by Arksey and O’Malley [35]. The review protocol was registered on Open Science Framework (OSF) (link: https://osf.io/2r8sj/).

### Identifying the research question

Given the review aim of mapping the existing evidence and identifying research gaps, this present study aims to address the following two research questions: (i) What are the sociocultural, emotional, and economic components that motivate intergenerational support in South Asian societies? and (ii) What are the intergenerational ambivalences that influence the health and well-being of older adults? The research questions were developed after a preliminary search of the existing literature and a discussion with the review team. To address the specific research gap in existing evidence, arrangements of intergenerational care, out-migration, and influence on health and well-being were the focus of the scoping review.

### Search strategy

A thorough review of the literature was conducted by using relevant keywords for both peer-reviewed and grey literature databases. We searched four databases from inception until February 2023, i.e. PubMed (NCBI), Scopus (Elsevier), Embase (Elsevier), and ProQuest (Clarivate). Google Scholar was used to identify grey literature. For this scoping review, we adopted the population, concept, and context (PCC) design. We used combinations of words of the main and related concepts of the PCC framework (see additional file 1 for search strategy on PubMed). The search string was modified according to the requirements of each database.

### Inclusion and exclusion criteria

Table 1 below shows the inclusion and exclusion criteria for selecting studies. We employed the following criteria to include studies at two stages (screening by title and abstract followed by full-text review): (1) only those studies published in the English language; (2) studies focussing on older adults (60 years and above) and were based on South Asian societies; (3) studies that focused on the issues of motivation or determinants of intergenerational relationships such as cultural, social, and financial support; (4) the intergenerational ambivalence in terms of out-migration, left behind, changing living arrangements, and conflict; and (5) subjective well-being of the older adults. The study focuses on the South Asian countries because of their shared cultural norms, strong filial responsibilities, and rapidly shifting family structures. In addition, the study excludes non-English studies to ensure accessibility, methodological rigour, and consistency in data analysis. However, the exclusion of local language could result in the omission of regionally specific caregiving practices and culturally nuanced viewpoints that are not documented in English-language research. Ethically, this study inadvertently overlooked insightful observations from non-English research.

**Table 1:** Inclusion and exclusion criteria

Framework	Inclusion criteria	Exclusion criteria
Population	Older men and women, 60 years and above	• Studies considering individuals aged 45–59 years as older adults • Studies focus on children, adolescents, and young people aged below 60 years
Concept	• Studies that focus on social, cultural, economic, and emotional attributes of intergenerational support • Studies focus on migration, left behind, loss of support, and conflict among young and older generations	We will not consider studies that solely focus on the influence of living arrangements, gender disparities, chronic diseases, economic status, and work participation on the well-being of older adults
Context	• South Asian countries • Articles having separate analysis for any of the South Asian country	Studies not conducted in the South Asian region
Type of studies	• Peer-reviewed journal articles (qualitative, quantitative, and mixed-methods design), theses, books, and book chapters	• All types of reviews, conference proceedings, reports, commentaries, editorials, newspaper articles, dissertations, articles unavailable, etc

### Screening and study selection

For this scoping review, relevant studies were included from quantitative, qualitative investigations, and mixed-methods studies that focus on the issues of intergenerational support and conflict that influence the subjective health and well-being of older adults. The screening was done based on the title, abstract, and full text by the first two authors. Any disagreement regarding the selection of articles was resolved by discussions among the authors and external experts.

### Data extraction: charting the data

The lead author extracted data on a pre-designed, pilot-tested data extraction chart, which was validated by the second author. The data extraction template was pilot tested on the first two selected articles, and then it was modified to include the sociocultural and economic support, conflict, and well-being of the older adults. Any disagreements at this stage were resolved by discussion with the corresponding author. The data extraction sheet included details of study characteristics such as author(s) name, year of publication, study design, study focus, study population group, determinants of intergenerational support, intergenerational ambivalence, subjective health and well-being, and key themes.

### Analysis: collating, summarising, and reporting results

Three key deductive themes were developed from the included studies using a thematic content analytic framework for presenting the ‘narrative account’ of existing literature [35]. Key themes were developed during the full article review process and charted in the characteristics table. This analysis has facilitated grouping the articles based on their content and key themes that dealt with determinants of intergenerational relations, intergenerational ambivalence, influence on life satisfaction and well-being, and relevance to the research question. In this study, narrative accounts of the articles that are charted in the characteristics table are presented in two ways. First, we analysed the nature, extent, and distribution of the studies selected for the scoping review. Secondly, the studies were categorised thematically to develop broader key themes.

## Results

### Extent and scope of existing literature

**Fig. 1:**
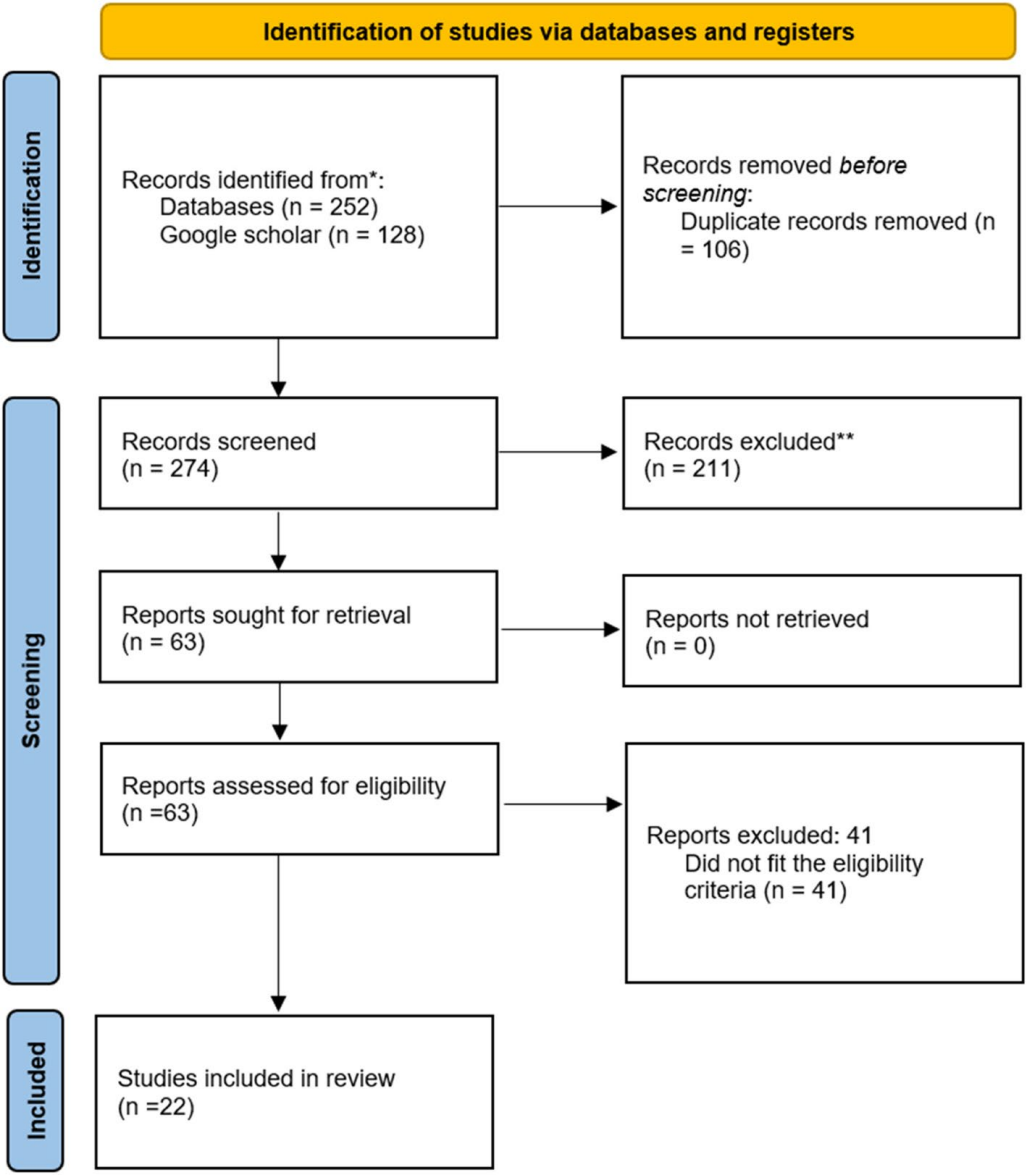
PRISMA flow diagram

A total of 380 studies were identified from database searches (*n* = 252) and Google Scholar (*n* = 128). The literature was exported to Rayyan software, and duplicates were resolved before the screening process. Titles and abstracts were screened using the predefined inclusion and exclusion criteria resulting in 63 studies for full-text review. Following a full-text assessment, 22 studies were selected for analysis [5, 24, 25, 29, 36–53]. The PRISMA flow diagram is presented in Fig. 1. Of the 22 studies, 5 were questionnaire survey-based quantitative studies, 15 were qualitative, and 2 were mixed methods. Surprisingly, no longitudinal studies were found in the study, thus limiting our understanding of the long-term effects of intergenerational support. There were 20 studies published in peer-reviewed journals, 1 was research thesis, and 1 was a book. In the course of analysing the geographical distribution of the studies, the following were noted: 15 were from India, 2 from Bangladesh, 3 from Nepal, 1 from Pakistan, and 1 from Sri Lanka. Looking through the lenses of social group and ethnicity, 10 studies were based on Hindu joint families (out of which 2 were specifically focused on Hindu upper caste in North India); 2 were mixed populations including Hindu, Muslim, Jain, and Christian religious groups; 2 were Sri Lankan Tamils; 1 was based on South Indian Christian; 1 focused on Bengali Muslims; and 6 studies did not mention the ethnicity or social group of the study population. There are gaps in regional comparisons because most studies concentrate on India, with little information coming from Bangladesh, Sri Lanka, and other South Asian countries. Though most of the studies are from India, this represents major cultural and traditional backgrounds in South Asia wherein living in a multigenerational joint family is common. Furthermore, four studies indicate a positive association between well-being and intergenerational support [26, 27, 31, 51], and three studies show intergenerational ambivalence by indicating emotional strain and caregiving stress [36, 43, 49]. The characteristics of included studies are detailed in Table 2.

**Table 2:** Study characteristics-intergenerational relations (*n* = 22)

Author(s) and year	Article title	Study design	Study setting	Study objectives	Key results
Kalavar et al., 2015 [36]	Transnational support of Asian Indian elderly in India: examining patterns of exchanges	Mixed-methods approach, focus group, and survey	Bangalore and Mumbai, India	Investigated the type of support exchanges between generations and the role of communication technology in the intergenerational support process	Transnational care is reciprocal in nature, and that communication technology has enhanced intergenerational contact
Dohlman et al., 2023 [37]	Generativity and engagement in grandparenting activities among older adults in northern Sri Lanka	Cross-sectional study, ordinary least squares (OLS) regression	Living in northern Sri Lanka, both urban and rural residents	The study explored the relationship between generative beliefs and specific grandparenting activity	Higher generative beliefs were positively associated with higher levels of engagement
Ahlin & Sen, 2020 [24]	Only near is dear? Doing elderly care with everyday ICTs in Indian transnational families	Ethnographic, participant observation, and interviews	Multi-sited fieldwork in Kerala	How information and communication technologies support in reformulation of care for older parents in India	ICTs bridge geographic gaps and make people feel as if they were in the same room even while physically apart
Gautam et al., 2011 [38]	Social interactions and depressive symptoms among community-dwelling older adults in Nepal: a synergic effect model	Cross-sectional face-to-face interview, multiple regression models	Older adults living in an urban area of Nepal	This study examines the synergic effects of intergenerational solidarity (emotional and instrumental support exchange and anticipated support) on depression of older adults	Emotional support exchange with the son diminished the positive association between conflict with the son and depression
Amin, Iftekhar, 2017 [25]	Perceptions of successful ageing among older adults in Bangladesh: an exploratory study	Interviews using in-depth semi-structured questionnaires	Bangladesh	To analyse older adults’ definitions of successful ageing in Bangladesh	Successful ageing is multidimensional such as adaptations to one’s changing body, financial security, religiosity and age identity, and social engagement
Niraula, Bhanu B., 1995 [39]	Old-age security and inheritance in Nepal: motives versus means	The data were taken from the Benighat Survey	Hill district of Central Nepal about 75 km south-west of Kathmandu	Synthesise expectations of old-age support in rural Nepal	Intergenerational transfer of property to the younger generation, especially among sons, provides a mechanism for old age support
Lamb, Sarah, 2005 [40]	Cultural and moral values surrounding care and (in)dependence in late life: reflections from India in an era of global modernity	Qualitative ethnographic fieldwork: interviews and observations, case studies	Old-age homes in and around Kolkata city	The study examines Indians’ perspectives on older care and the significant changes by exploring how beliefs and practices speak about deep embedded cultural-moral visions of the relationship	The NGOs and government should support social security for older adults. Besides, the older adults should also change their mind-set about their children that they will provide support in their old age
Chandra Sekaran et al., (2021) [41]	‘No, you should not beat our child because he will become aggressive:’ applying a multi-method approach to explore intergenerational transmission of parenting practices	Qualitative methods: in-depth interviews and observations, case studies. Thematic approach was used to interpret the results	The study was conducted at Udupi and Brahmavara taluks of Udupi, a coastal district in southern India	To explore and gain insight into the parenting experiences of participants from two generations and the possible transmission across the two generations	Family has been the central to Indian society and interdependence, and dyadic relations between older parents and adult children were fundamental to intergenerational relations
Jothikaran et al. (2020) [42]	Older adults in traditional and modern living arrangements in southern India: the importance of maintaining a sense of belonging and positive intergenerational exchanges	The study adopted qualitative approach	Older participants living in the same household with married children in two South Indian states	To explore the perspectives and everyday experiences of older persons living in different arrangements in India	Older adults perceive social, cultural, and emotional attachment as long as they feel that their offspring adhered to traditional values
Goldstein et al., 1983 [43]	Social and economic forces affecting intergenerational relations in extended families in a third-world country: a cautionary tale from South Asia	Qualitative: An open-ended in-depth interview was conducted	Bistatol neighbourhood of Kathmandu in Nepal	To explore the impact of social and economic changes on the family and elderly people in urban Nepal	The impact of changing economic and filial structure deeply influenced the care system; older parents are not getting love, attention, and respect from their children
Jothikaran et al., 2023 [44]	Views and experiences of adult children concerning intergenerational relationships with their older kin: a qualitative study from South India	Qualitative interpretative approach	Two of the South Indian states (one a well-performing state, Tamil Nadu, and the other a poorly performing state, Telangana)	To explore the experiences of adult children concerning intergenerational relationships with their parents with regard to reciprocity of care and support and the challenges they experience and strategies they adopt to overcome those challenges	Despite changes in work life and complexities, they seem to uphold the cultural values and norms related to providing intergenerational care and support
Abrar et al., 2018 [45]	The emerging need of elder’s institutional care in Pakistan	Qualitative thematic approach: data was collected through semi-structured interview for the collection of in-depth information	Peshawar city for families having the oldest-old family members and Gulbahar town in Pakistan	To establish an alternative care or support mechanism for older adults in Pakistan	There is a growing need to establish an alternative care or support mechanism for the welfare of these socially vulnerable inhabitants in the country
Kalavar & Jamuna, 2008 [46]	Interpersonal relationships of elderly in selected old-age homes in urban India	Qualitative: face-to-face in-depth interviews	‘Pay and stay’ homes in the four cities of Hyderabad, Bangalore, Chennai, and Thiruvananthapuram	To understand the relocation experience of older adults who reside in the pay and stay homes and nature of interpersonal relationships	The social landscape of India is changing. With the rapid proliferation of ‘pay and stay’ homes in urban India, it is clear that there is a movement toward this arrangement
Bardhan, A. (2022) [37]	Family care and its impact on the life of elderly people: a study in Dhaka city	Qualitative: case study method and in-depth interviews, two focus-group discussions	Dhaka city of Bangladesh	The main objective of the study was to explore and understand the relationship between the challenges of elderly people and the nature of family care towards them	The main challenges of later life are poverty, ageism, and abuse of elderly people, which affect intergenerational relation particularly financial support
Chandra, 2017 [53]	Mending Maya: an analysis of ageing and intergenerational connection in Delhi, India	Qualitative: semi-structured interview	Living in an old-age home in Delhi, India	To examine intergenerational connection and understanding and test the effectiveness of implementing an art-based intergenerational intervention called Mending Maya	Intergenerational relations in India are more likely to be successful if it builds on cultural values that emphasise family and community
Gangopadhyay, 2021 [47]	Culture, context, and ageing of older Indians: narratives from India and beyond	Qualitative: semi-structured interview, observations	Older adults living in multigenerational families, old-age homes, widowed older adults staying with their unmarried adult children	To provide an insight into various intricacies and dynamics associated with intergenerational relationships	The older residents continued to expect emotional support from their adult sons despite these expectations, and the older adults had shifted to institutional care home and were putting in their efforts to adjust to this home
Verma et al., 2022 [48]	Experience of well-being: a cross-generational study in India	A cross-sectional correlation design	Gorakhpur city and Reotipur village located in Utter Pradesh	To investigate the patterns of intergenerational relations (social support, social relations, and autonomy) in Indian family and its role on their subjective well-being (happiness)	Level of satisfaction has been found to be clearly correlated with the level of perceived social support, social relations, and autonomy
Ugargol & Bailey, 2021 [5]	Reciprocity between older adults and their caregivers in emigrant households of Kerala, India	Qualitative study: semi-structured in-depth interview and observations	Kottayam district of Kerala, a southern Indian coastal state	To examine how left-behind older adults and their family caregivers recognise, interpret, and give meaning to reciprocal exchanges, expectations, and obligations in their care relationship	Non-reciprocity in the exchange relationship threatened the relationship and often led to frustrations and friction between the older adult and the caregivers, thus compromising the wellbeing
Mishra & Kaur, 2021 [29]	Gender imbalance, marriage squeeze, and multiple biological clocks: exploring challenges to the intergenerational contract in North India	Qualitative methods: ethnographic fieldwork-semi-structured interviews	Five villages of Sonipat and Hisar districts of Haryana	To explore the changes in the relationship between matured unmarried sons and their ageing parents	The essence of intergenerational contract is that parents are obligated to invest in the upbringing, education, and well-being of their children. In turn, children are expected to return the favour by taking care of their parents as they grow older and become dependent
Gangopadhyay & Samanta, 2017 [49]	Family matters: ageing and the intergenerational social contract in urban Ahmedabad, Gujarat	In-depth qualitative interviews and observations	The city of Ahmedabad, Gujarat, India	To understand the complex interactions of family and intergenerational relationships in an emerging city in India	Though family cohesion was maintained in intergenerational relationships, ambivalence too surfaced as an emotional dimension
Ungar & Mahalingam, 2003 [50]	‘We're not speaking any more’: a cross-cultural study of intergenerational cut-offs	Mixed-methods approach: survey methodology, focus groups	Silver Innings Foundation and Non-Resident Indian Parent Association (NRIPA) in Bengaluru and Mumbai, India	To investigate the type of support exchanges between generations and the role of communication technology in this process	Transnational care is reciprocal in nature, and that communication technology has enhanced intergenerational contact
Bawdekar & Ladusingh, 2012 [51]	Intergenerational time and monetary support among urban Indian families	Quantitative survey data: two-stage least square (2SLS) regression analysis	One-hundred sixty-two wards in the Pune Municipal Corporation	To examine the nature and pattern of time transfers between co-residing and non-co-residing parents and their adult married children in an urban setting in India	Time support is more frequent and intense when parents reside in the same city, whereas monetary transfers dominate when parents reside in a distant city or village

### Arrangements of intergenerational support

In this present study, emotional, cultural, social, and financial dimensions of intergenerational support have been grouped into intergenerational arrangements and address the research question. Traditionally, in the South Asian cultural context, adult children provide instrumental, emotional, and financial support for their parents with reverence. Several studies highlighted that not only financial support but also food, clothing, shelter, love, respect, and staying close to children and grandchildren are integral parts of intergenerational family support [36, 40, 42].

### Emotional care and intergenerational relations

Out of 22 included studies, 15 studies have revealed that exchange of emotional and instrumental support among older parents and adult children motivates the intergenerational solidarity [24, 25, 29, 36, 38, 40, 42–44, 46–49, 52, 53]. Moreover, positive emotional changes enable to resolve the conflicts among family members [29]. While analysing the gendered nature of emotional care, older adults receive higher levels of emotional support from their sons, whereas adult children are largely dependent on their mothers. Another study by Bawdekar and Ladusingh argued that older parents have strong emotional attachments, particularly with daughters, which influence care in later life [51].

### Sociocultural support and intergenerational relations

Religion-based cultural norms are profoundly associated with intergenerational caregiving expectations. Almost all the included studies have stated that older adults are culturally valued and expected to respect and take care of older parents across the South Asian context. For instance, Ugargol and Bailey highlighted that strong reciprocal filial obligations exist in India and are culturally effectuated through intergenerational co-residence [5]. Some other studies revealed that grandparents’ role in raising their grandchildren is highly respected and helps preserve Indian culture, religion, and heritage [40, 51]. Similarly, in Bangladesh, it is expected that families and communities will provide care for their older members [52]. In addition, caring for older parents is a social norm for adult children, and not fulfilling the care roles is considered as shame for them [38, 41]. Some of the studies also focused on social factors such as respect for older adults for their experience, wisdom, knowledge, and skills to determine the intergenerational exchanges [44]. In contrast, moving older parents into institutions is a social stigma against the family in India [36] and Bangladesh [25, 52].

### Financial support and intergenerational relations

Financial support is the most significant element of the reciprocal exchange of intergenerational relations between sons and parents. About 15 studies have mentioned that when parents grow older and become more dependent, their children provide them with financial care and support in return across South Asian societies [5, 24, 25, 38–40, 42, 44–47, 49–51, 53]. Niraula (1995) argued that intergenerational transfer of property through inheritance from the older to the younger generation, especially among sons, provides a mechanism for old-age support [39]. Jothikaran and colleagues stated that older parents transfer their economic resources to their children, and in exchange, they expect their children to support their economic expenses in their later life [42]. However, economically less-advantaged young adults perceive economic support to older parents as a burden which aggravates their care-related challenges.

### Challenges of intergenerational arrangements and ambivalence

Traditionally, older adults were taken care of by the immediate family members, but due to out-migration, urbanisation, and modernisation of society changing living arrangements, culture, and values, conflicts are emerging in the intergenerational relations, thus influencing the solidarity. A total of 12 studies revealed that migration of an adult child impacts intergenerational expectations, which in turn alters family dynamics [5, 25, 36, 38, 42, 45–50, 52]. Expectations of family life are disrupted and renegotiated as a result of transnational migration [5, 36, 45] further state that the migration of young children produces a sense of being ‘left behind’ among older parents. In contrast, in a study conducted in Kerala, India, the authors argued that older parents consider migration as a ‘family project’ and a source of economic security for later life [24].

The results also demonstrate that conflict between older parents and young children, due to outmigration and subsequent changing family dynamics, is another dimension of intergenerational ambivalence. Among 22 studies, 11 studies revealed that intergenerational conflict leads more and more older people to shift into old-age homes, particularly in urban areas [5, 25, 29, 38, 43, 46, 47, 49, 51–53]. The major reasons for conflict were the absence of daytime caregivers, failing health, fear of crime, and children living overseas [41, 45]. In addition, limited communication between older parents and adult sons, transfer of property, and lack of financial care also led to conflict across South Asian societies [46, 47].

### Changing living arrangements and contradictions

The studies revealed that there is an overwhelming preference for co-residence with the son, whereas living with other relatives is considered a misfortune. Jothikaran and colleagues argued that co-residence with adult children in the same household strengthens intergenerational exchanges [42]. Living arrangements of older adults obligate daughters-in-law to provide care to parents-in-law in the Indian context, but migration of children and grandchildren is increasingly changing the living arrangements of the older parents, sometimes coercing them to live independently with or without a spouse [29, 39, 49]. Conversely, it is argued that though out-migration declines solidarity, grandparents continue to act as caregivers in co-residential living arrangements [51].

### Affect and importance for health and well-being

Our review found no studies that measured objective well-being in connection with intergenerational support. Hence, we focused on subjective health and well-being indicators such as stress/depression, loneliness/isolation, loss of support, and life satisfaction. The results suggest that the declining joint family system, the rise of women’s work participation, changing attitudes of the younger generation towards older adults, possible decreasing value system, and subsequent neglect of older parents have posed challenges to long-term care provision to older adults. Moreover, the out-migration of young adults creates a care gap and fear of living alone and negatively influences the psychological health of the older adults [36, 38]. There were eight studies, which highlighted that loss of support to older adults, caused by intergenerational ambivalence including out-migration of children, demise of spouse, nuclear family, and moving to old-age homes, produce a sense of neglect among the older parents [38, 40, 43–45, 47, 48, 52]. The older adults also perceived that they would lose respect and social status if they lost support from children. The impact of loss of care from children is reflected in the deteriorating mental health of older adults [48].

Older adults are often reported to experience feelings of isolation, loneliness, and hopelessness when their adult children migrate [5, 24, 25, 36, 42, 44, 48, 51]. The absence of the emigrant child appears to have a negative effect on the parent’s psychological well-being, including evidence of loneliness. For instance, negative emotional relations with adult children lead to conflict and contribute to the association with depressive symptoms among older adults in Nepal [39]. In contrast, emotional support from and to the son buffered the negative consequences of conflict on depression among older adults which result in lower levels of depression [43, 47, 53]. In addition, the older parents who moved to institutional care centres, also known as old-age homes in South Asia, feel lonely because they perceive they are being left out in the old-age homes. On the other hand, there is a debate that older adults living in multigenerational houses are under stress due to a lack of privacy, difficulty adapting to other household members’ behaviour, and declining control over the use of their spaces within the home [47].

Living with sons, extended families, and households, which are ideally ‘expected to fulfil the duty of caring for older parents’, is believed to improve social and economic security and a higher level of satisfaction in later life [37, 43]. The majority of the studies revealed that living alone was associated with low subjective well-being, and those living with immediate family members reported improved general well-being [25, 39, 40, 52]. Older adults are more satisfied with the care of children in the family than living with extended family members and old-age homes [5]. In traditional arrangements, different forms of support are offered, ranging from listening to the emotional distress to being physically present, satisfying the older parents in their everyday care and support [49].

Those older adults living in old-age homes experienced that the absence of family members at the old-age home was a major source of dissatisfaction [46, 51]. Older people living in old age homes did not get respect, love, and affection from their family members and were highly dissatisfied [5, 48, 50, 53]. Older parents felt sad due to changes in living arrangements [49]. They perceived that they were being dumped at the old-age homes by their family members. In contrast, older parents who receive support from their children often feel a loss of autonomy and stress resulting in lower levels of subjective well-being [38, 48]. Residing in a co-residence has the advantage of getting time and financial support from the children. However, the dwindling family structure adversely affects the level of satisfaction and well-being of the older parents [51].

## Discussion

This scoping review has shown that intergenerational support arrangements are shaped by social and cultural contexts across South Asia, and that exchange and reciprocity remain the motivation for providing intergenerational support. Intergenerational support manifests itself mainly through financial, emotional, and instrumental support in shared households. However, the intergenerational conflict including out-migration of young children and changing living arrangements leads to loss of support and produces a sense of neglect and loneliness, which affect the psychological health and subjective well-being of older adults.

The results suggest that there are gaps in empirical evidence of how intergenerational arrangements, conflict, and ambivalence dimensions affect the physical health and objective well-being of older adults. Existing studies predominantly emphasise qualitative assessments of intergenerational support, often overlooking the critical dimension of objective well-being. Further, studies often overlook the migratory history and experiences of older individuals and how these factors impact their health and care requirements as they advance to later age. Moreover, there is a scarcity of longitudinal studies that provide extended data availability and a dynamic view of ageing-related changes. Future endeavours should focus on objective indicators such as physical health, income, education, employment, social group, and status, to explore the multifaceted connections between intergenerational support and individuals’ tangible quality of life. This scoping review also indicates that a significant body of existing literature focused on intergenerational transfer and support from patriarchal perspectives, wherein the focus has been on property transfer from older parents to sons and the ensuing care provided by sons. However, a notable gap exists in understanding intergenerational support from the viewpoint of women, especially concerning gender disparities in health and subjective well-being. There is a lack of evidence on how out-migration and conflict impact widowed older women living with or without spouses. In addition, the gender differences in caregiving responsibilities have been overlooked in existing research. In South Asian patriarchal family system, the care responsibilities are shouldered on women, such as daughters and daughters-in-law, compared to sons. Further research is required to examine how these gendered caregiving roles change over time, particularly in light of shifting family dynamics and migration. The scoping review also indicated that there is a lack of studies focusing on the role of technology particularly for those domestic or transnational migrants who provide distant care to their older parents back home. Digital tools such as telemedicine, mobile banking, and video calls have become essential resources for distant caregiving. These technologies facilitate reducing emotional distance, providing financial support, and enabling remote healthcare access, thus partially compensating for the physical absence of migrant children.

Another important research gap that arose from the scoping review is the lack of policy research. Hardly any study focused on the policy frame for intergenerational support, the majority of the studies documented how dwindling joint family relations affect subjective well-being. In this context, adopting the Madrid International Plan of Action on Ageing is significant for these South Asian societies to promote intergenerational solidarity. The Madrid International Plan of Action on Ageing states that generational solidarity at all levels — in families, communities, and nations — is essential for achieving a society for all ages. However, India and South Asia face significant challenges in meeting the MIPAA priority areas and Sustainable Development Goals (SDGs) related to ageing. While some progress is evident, gaps in social protection, healthcare, and support systems persist, especially concerning older adults in rural areas and those affected by migration. Hence, policies around intergenerational support would promote the values to uphold through acknowledgement and incentives such as Singapore’s intergenerational bonding efforts. South Asian countries with the advent of ageing societies need active intergenerational support from the state. The policies should promote social protection and antipoverty schemes including pensions, social security, conditional cash transfers, and subsidised guardianships adhering to MIPAA. Besides tax relief to taxpayers if they are financially supporting parents and grandparents, intergenerational care policies, such as allowances for grandparents to care for their grandchildren, have the potential to strengthen this process.

The results suggest that past intergenerational family relations emphasised norms and values and were marked by the great respect and obedience of the younger generations to the authoritative older generations [25, 36, 40]. Studies on other Asian societies such as Taiwan and Malaysia also found that there is intergenerational support among family members about living arrangements and material or financial support [54, 55]. However, the cultural-moral conceptions of caring and intergenerational relationships are extremely subjective and may not be adequately conveyed in scales or direct questions in a society where structured (generational) dependency is socially valued [22]. The results also revealed that emotional support exchange with the son diminished the positive association between conflict with the son and depression. The finding is consistent with the findings of Chen and Feeley who argued that social and emotional support from spouse or partner, children, and friends or relatives reduced loneliness and improved well-being among older adults in the United States [56]. In this context, when older adults prefer to live with children and other family members in a multigenerational household, ‘ageing in place’ is recommended for caring for older adults as long as possible. In South Asian societies where most older adults are dependent on their children and other family members for financial support, intergenerational resources transfer in terms of land and house motivates the young generation to maintain intergenerational solidarity. Within the reciprocity exchange model, older adults are important financial support providers within their family network [57]. However, research from Western societies suggests financial transfers are influenced by intergenerational relationships, such as emotional closeness [58].

South Asian societies, which were traditionally multigenerational, are increasingly adopting Western family structures and losing filial piety to obtain family support [59]. There is evidence that intergenerational relations are undergoing transitions due to social and cultural changes; teenagers especially perceive their grandparents differently [45, 53]. Croll also argued that urbanisation, the new lifestyle in a modern urban world, and the aspirational migration of young children have engendered individualism and damaged filial obligation [18]. Several social surveys and interpretive studies throughout Asia revealed that conflicts and stresses due to the widening generation gap have engendered diminishing respect and care for older adults [60–63]. Different ethnographic studies across Asia have also established the increasing gap between the generations due to the effect and adaptation of new values, attitudes, and behaviours of a more self-determining younger generation [18]. Similarly, several social survey studies found that India, like many other South Asian Societies, has also experienced a declining multigenerational joint family and increasing nuclear family system that led to a growing care gap due to increasing attitudes of young generation to live separately [64–66]. Once considered to be the sole responsibility of the family members in general and the women in the family in particular, the whole scenario of care has changed into a matter of serious concern among the young generation.

The findings demonstrated that intergenerational relationships and family social support have a direct impact on older parents’ mental health. A positive intergenerational relationship, such as meaningful communication between older people and their adult children, can reduce symptoms of loneliness and despair and improve the mental health of older adults [36, 38, 49]. Conversely, a conflicting intergenerational relationship between older adults and children weakens the well-being of older people and causes depression [25, 67]. Therefore, intergenerational relationships are critical for the mental health and subjective well-being of older adults. This finding is consistent with the findings of Golden and colleagues [68] and Zhou et al. [69] who noted that the wider the older adults’ family network, the more opportunities for the older people to speak and interact with family members, reducing their sensation of loneliness. On the other hand, we found that out-migration has an adverse impact on the subjective well-being of the older adults who perceived they are being ‘left behind’. Out-migration of adult children has a severe impact on their older parents, causing loneliness, isolation, and a loss of fundamental assistance [70, 71]. Antman reported that the migration of adult children is adversely associated with the physical and mental health of older parents [72]. Similarly, the mental health of older parents was found to deteriorate after the migration of children to China and South Africa [73–75]. The evidence for Thailand is more mixed, where Adhikari et al. reported a negative association [76] and Abas et al. found the opposite [77].

### Limitations of the study

It is important to acknowledge that our analysis has certain limitations stemming from the exclusion of studies conducted in other and/or local languages in our review. These studies might offer valuable insights and perspectives that could enrich our understanding of intergenerational support. Furthermore, it is plausible that there exist additional reports and studies, particularly those published in newspapers, magazines, newsletters, and review studies, which were not captured in our review. These omissions have potentially introduced biases or gaps in our analysis, as these sources present unique cultural, social, or demographic nuances that are not adequately represented by the studies included in our research. In addition, there might be quantitative studies that focus on family support variables but do not relate to intergenerational support; those studies have not been included in our analysis. Moreover, the disordered intergenerational relations and out-migration may have a positive influence on the life satisfaction and well-being of the older parents in other geographical regions. Furthermore, the remittance may have a positive impact on the mental health and well-being of older adults which has not been captured in this study.

## Conclusions and recommendations

The study has provided a comprehensive overview of the relationship between intergenerational support and its impact on health and well-being. It highlighted existing support types and their social and cultural embeddedness and how this comes increasingly under pressure. Through an extensive review of existing literature, various types of studies were analysed, encompassing narrative and cross-sectional studies predominantly due to the limitations in longitudinal data availability. This approach allowed for a comprehensive assessment of the current state of knowledge regarding intergenerational support and its effects on health indicators across South Asian geographical contexts. This review study has illuminated a critical gap in the existing literature regarding the interplay between intergenerational support and its impact on the health and well-being of older adults. Despite the undeniable importance of strong family networks and cross-generational relationships, particularly in the context of South Asian countries experiencing a demographic shift towards an ageing society, limited attention has been devoted to this significant aspect of older adults’ well-being. The scarcity of comprehensive studies examining the multifaceted dynamics of intergenerational support and its effects on the health and well-being of older adults highlights a missed opportunity for enhancing the quality of life for ageing populations, particularly in regions like South Asia where these dynamics carry cultural, social, and economic significance. By delving into existing evidence, this study aims to contribute to a deeper understanding of the multifaceted dimensions of ageing and intergenerational dynamics. However, the overrepresentation of Indian studies, which restricts the findings’ generalizability throughout the region, emphasises the need for further research from other South Asian countries such as Bangladesh, Sri Lanka, and Nepal. In addition, the overdependence on qualitative and cross-sectional studies limits our understanding of long-term caregiving dynamics which emphasise the significance of further longitudinal study. These limitations highlight the necessity of more comprehensive regional and methodical approaches in future research and policy recommendations.

The implications of this research reach beyond academic discourse and extend into policy formulation and practical interventions. In this context, the study aligns with the priority areas outlined in the Madrid International Plan of Action on Ageing (MIPAA) to shed light on the intersection of intergenerational relationships and the promotion of well-being. As MIPAA emphasises the priority areas of social participation, care and support, and income security for older adults, it is imperative to recognise the potential role of intergenerational support systems in addressing these objectives. While South Asian nations such as India have made progress in addressing MIPAA priority areas, there are still substantial challenges to overcome. In addition to other initiatives like pension plans and healthcare programmes like the National Programme for Health Care of the Elderly (NPHCE), India has enacted the National Policy on Older Persons. But there are still gaps in family-based care, social security, and access to healthcare, particularly in rural areas. Therefore, in India, the expansion of government-funded pension policies and community-based elder care facilities will benefit older adults, particularly in low-income urban and rural communities, whereas Sri Lanka could strengthen its universal healthcare system by integrating mental health and geriatric care. Similarly, Bangladesh, Pakistan, and Nepal need to develop formal caregiver training programmes and provide tax incentives for family caregivers. However, policies including pension plans and elder care initiatives have been implemented in Bangladesh, Nepal, and Sri Lanka; nevertheless, development is being impeded by financial constraints and differences between urban and rural areas. Therefore, strengthening social protection and healthcare systems is essential for further advancements. The governments need to expand policies that encourage community-based older care services, especially for people who do not have family caregivers. Providing caregiver allowances or tax breaks to family caregivers would reduce their financial burden. In addition, government and non-government organisations (NGOs) should promote, through public awareness campaigns, the use of digital tools such as telemedicine, online mental health support, and mobile banking to enable distant caring and financial support to older adults with migrant children.

This study also recommends that intergenerational family support serves as the primary, and sometimes the sole, source of assistance and care for older adults. To effectively support older adults, ageing societies must prioritise two fundamental aspects: first, the continuous expansion and refinement of knowledge related to family dynamics and their impact on older adults’ lives, which will inform evidence-based policies and interventions, and, second, the creation of living environments and support arrangements that facilitate and strengthen family caregiving roles while also ensuring that families receive the necessary resources and assistance. This holistic approach is essential to enhance the quality of life and well-being of older adults in ageing societies with limited public support systems.

## Supplementary Information

Additional file 1: Search strategy.

## Data Availability

Data will be made available on request to corresponding author.

## References

[1] Quashie NT. Who supports whom? Gender and intergenerational transfers in post-industrial Barbados. J Cross Cult Gerontol. 2015;30:189–216. 10.1007/s10823-015-9260-2. 25894849 10.1007/s10823-015-9260-2

[2] Wangliu Y. Does intergenerational support affect older people’s social participation? An empirical study of an older Chinese population. SSM Popul Health. 2023;22:101368. 10.1016/j.ssmph.2023.101368. 36873267 10.1016/j.ssmph.2023.101368 PMC9974446

[3] Katz R, Lowenstein A. Theoretical perspectives on intergenerational solidarity, conflict and ambivalence. Ageing and Intergenerational Relations: Bristol University Press; 2010. p. 29–56. 10.46692/9781847422064.004.

[4] Albertini M, Kohli M, Vogel C. Intergenerational transfers of time and money in European families: common patterns — different regimes? J Eur Soc Policy. 2007;17:319–34. 10.1177/0958928707081068.

[5] Ugargol AP, Bailey A. Reciprocity between older adults and their care-givers in emigrant households of Kerala. India Ageing Soc. 2021;41:1699–725. 10.1017/S0144686X19001685.

[6] Herrera MS, Galkuté M, Fernández MB, Elgueta R. Grandparent-grandchild relationships, generativity, subjective well-being and self-rated health of older people in Chile. Soc Sci Med. 2022;296:114786. 10.1016/j.socscimed.2022.114786. 35151151 10.1016/j.socscimed.2022.114786

[7] Gallardo-Peralta LP, de Roda ABL, Ángeles Molina- Martínez M, Schettini del Moral R. Family and community support among older Chilean adults: the importance of heterogeneous social support sources for quality of life. J Gerontol Soc Work 2018;61:584–604. 10.1080/01634372.2018.1489928. 10.1080/01634372.2018.1489928 29979944

[8] Araos C, Siles C. “Juntos pero no revueltos”: family residential dependence and care vulnerabilities along the life course. Adv Life Course Res. 2021;49:100404. 10.1016/j.alcr.2021.100404. 36695117 10.1016/j.alcr.2021.100404

[9] Lüscher K, Hoff A. Intergenerational ambivalence: beyond solidarity and conflict. Intergenerational Relations: Bristol University Press; 2013. p. 39–64. 10.46692/9781447300991.004.

[10] Suitor JJ, Gilligan M, Pillemer K. Conceptualizing and measuring intergenerational ambivalence in later life. J Gerontol B Psychol Sci Soc Sci. 2011;66B:769–81. 10.1093/geronb/gbr108. 10.1093/geronb/gbr108 PMC3198248 22002969

[11] Thapa DK, Visentin D, Kornhaber R, Cleary M. Migration of adult children and mental health of older parents ‘left behind’: an integrative review. PLoS ONE. 2018;13:e0205665. 10.1371/journal.pone.0205665. 30346972 10.1371/journal.pone.0205665 PMC6197646

[12] Jayakody R, Thornton A, Axinn W. Perspectives on international family change. In: Jayakody R, Thornton A, Axinn W, editors. International Family Change: Ideational Perspectives. 1st ed. New York: Routledge; 2007. p. 1–18. 10.4324/9780203809648.

[13] Desai V, Tye M. Critically understanding Asian perspectives on ageing. Third World Q. 2009;30:1007–25. 10.1080/01436590902959263.

[14] Jayawardhana T, Anuththara S, Nimnadi T, Karadanaarachchi R, Jayathilaka R, Galappaththi K. Asian ageing: the relationship between the elderly population and economic growth in the Asian context. PLoS ONE. 2023;18:e0284895. 10.1371/journal.pone.0284895. 37093815 10.1371/journal.pone.0284895 PMC10124889

[15] Kaluthantiri KDMS. Ageing and the changing role of the family in Sri Lanka. University of Adelaide, 2015.

[16] Rajwar E, Pundir P, Challa S, Prince AM, Parsekar SS, Vogt T. A scoping review of income support programs offered to older adults living in South Asian countries between 2000 and 2021. Health Soc Care Community. 2024;2024:1–19. 10.1155/2024/1711756.

[17] Ashiq U, Abbas N. Dwindling classical cultural trends of family: prospect consequences on elderly care in modern Pakistan. Orient Research Journal of Social Sciences. 2020;5:1–11.

[18] Croll EJ. The intergenerational contract in the changing Asian family. Oxford Development Studies. 2006;34:473–91. 10.1080/13600810601045833.

[19] Guo Q, Gao X, Sun F, Feng N. Filial piety and intergenerational ambivalence among mother–adult child dyads in rural China. Ageing Soc. 2020;40:2695–710. 10.1017/S0144686X19000783.

[20] Liu J. Filial piety, love or money? Foundation of old-age support in urban China. J Aging Stud. 2023;64: 101104. 10.1016/j.jaging.2023.101104. 36868617 10.1016/j.jaging.2023.101104

[21] Park NS, Chiriboga DA, Chung S. The effect of social capital and family support on loneliness among Korean adults: intergenerational differences. J Intergener Relatsh. 2021;19:109–23. 10.1080/15350770.2021.1868239.

[22] Samanta T. The joint family and its discontents: interrogating ambivalence in intergenerational relationships. Asian Popul Stud. 2019;15:28–46. 10.1080/17441730.2018.1560659.

[23] Zhang M, Lin T, Wang D, Jiao W. Filial piety dilemma solutions in Chinese adult children: the role of contextual theme, filial piety beliefs, and generation. Asian J Soc Psychol. 2020;23:227–37. 10.1111/ajsp.12395.

[24] Ahlin T, Sen K. Shifting duties: becoming ‘good daughters’ through elder care practices in transnational families from Kerala, India. Gend Place Cult. 2020;27:1395–414. 10.1080/0966369X.2019.1681368.

[25] Amin I. Perceptions of successful aging among older adults in Bangladesh: an exploratory study. J Cross Cult Gerontol. 2017;32:191–207. 10.1007/s10823-017-9319-3. 28523474 10.1007/s10823-017-9319-3

[26] Shyama C. Direct and indirect income support and their determinants: developing an income profile for older adults in Sri Lanka. De Silva, Tiloka. 2022;30:463–87.

[27] Guo M, Aranda MP, Silverstein M. The impact of out-migration on the inter-generational support and psychological wellbeing of older adults in rural China. Ageing Soc. 2009;29:1085–104. 10.1017/S0144686X0900871X.

[28] Liu C, Zhou S, Bai X. Intergenerational relationship quality, sense of loneliness, and attitude toward later life among aging Chinese adults in Hong Kong. Front Psychol. 2022;13:930857. 10.3389/fpsyg.2022.930857. 36017420 10.3389/fpsyg.2022.930857 PMC9397484

[29] Mishra P, Kaur R. Gender imbalance, marriage squeeze and multiple biological clocks: exploring challenges to the intergenerational contract in North India. Anthropology & Aging. 2021;42:97–111. 10.5195/aa.2021.251.

[30] Executive Committee of the High Commissioner’s Programme; Standing Committee. UNHCR’s Policy on Older Refugees, EC/50/SC/CRP.13, Annex II, UN High Commissioner for Refugees (UNHCR). 2000.

[31] Gorman M. Development and the rights of older people. In: Randal Judith, German T, Ewing D, editors. The Ageing and Development Report. Poverty, Independence and the World’s Older People. 1st ed., London: Earthscan Publications Ltd; 1999, p. 0–19.

[32] Solar O, Irwin A. A conceptual framework for action on the social determinants of health. Social Determinants of Health Discussion Paper 2 (Policy and Practice). . Geneva: 2010.

[33] Fingerman KL, Kim K, Tennant PS, Birditt KS, Zarit SH. Intergenerational support in a daily context. Gerontologist. 2016;56:896–908. 10.1093/geront/gnv035. 26035892 10.1093/geront/gnv035 PMC5019045

[34] Tricco AC, Lillie E, Zarin W, O’Brien KK, Colquhoun H, Levac D, et al. PRISMA Extension for Scoping Reviews (PRISMA-ScR): checklist and explanation. Ann Intern Med. 2018;169:467–73. 10.7326/M18-0850. 30178033 10.7326/M18-0850

[35] Arksey H, O’Malley L. Scoping studies: towards a methodological framework. Int J Soc Res Methodol. 2005;8:19–32. 10.1080/1364557032000119616.

[36] Kalavar JM, Zarit SH, Ferraccio BJ. Transnational support of Asian Indian elderly in India: examining patterns of exchanges. Care Manag J. 2015;16:141–9. 10.1891/1521-0987.16.3.141. 26363157 10.1891/1521-0987.16.3.141

[37] Dohlman CS, Zalla LC, Chung EO, Østbye T, Maselko J. Generativity and engagement in grandparenting activities among older adults in northern Sri Lanka. The International Journal of Aging and Human Development. 2023;97:249–62. 10.1177/00914150221143955. 36475899 10.1177/00914150221143955

[38] Gautam R, Saito T, Houde SC, Kai I. Social interactions and depressive symptoms among community dwelling older adults in Nepal: a synergic effect model. Arch Gerontol Geriatr. 2011;53:24–30. 10.1016/j.archger.2010.06.007. 20598380 10.1016/j.archger.2010.06.007

[39] Niraula BB. Old age security and inheritance in Nepal: motives versus means. J Biosoc Sci. 1995;27:71–8. 10.1017/S002193200000701X. 7876297 10.1017/s002193200000701x

[40] Lamb S. Cultural and moral values surrounding care and (in)dependence in late life: reflections from India in an era of global modernity. Care Manag J. 2005;6:80–9. 10.1891/152109805780650742. 16544869 10.1891/cmaj.6.2.80

[41] Chandra Sekaran V, Bailey A, Kamath VG, Ashok L, Ravindran SK, Kamath A, et al. ‘No, you should not beat our child because he will become aggressive:’ applying a multi-method approach to explore intergenerational transmission of parenting practices. PLoS ONE. 2021;16:e0258306. 10.1371/journal.pone.0258306. 34618867 10.1371/journal.pone.0258306 PMC8496842

[42] Jaihind Jothikaran TA, Meershoek A, Ashok L, Krumeich A. Older adults in traditional and modern living arrangements in southern India: the importance of maintaining a sense of belonging and positive intergenerational exchanges. J Aging Stud. 2020;54:100867. 10.1016/j.jaging.2020.100867. 32972624 10.1016/j.jaging.2020.100867

[43] Goldstein MC, Schuler S, Ross JL. Social and economic forces affecting intergenerational relations in extended families in a Third World country: a cautionary tale from South Asia. J Gerontol. 1983;38:716–24. 10.1093/geronj/38.6.716. 6630908 10.1093/geronj/38.6.716

[44] Jaihind Jothikaran TA, Meershoek A, Ashok L, Krumeich A. Role of spiritual experiences in shaping the quality of intergenerational relationships - exploring views of older adults in South India. J Relig Spiritual Aging 2023:1–18. 10.1080/15528030.2023.2259824.

[45] Abrar M, Riaz S, Alam H. The emerging need of elder’s institutional care in Pakistan. New Horiz. 2018;12:45–58.

[46] Kalavar JM, Jamuna D. Interpersonal relationships of elderly in selected old age homes in urban India. Interpersona: An International Journal on Personal Relationships. 2008;2:193–215. 10.5964/ijpr.v2i2.26.

[47] Gangopadhyay J. Culture, context and aging of older Indians. Narratives from India and Beyond. Singapore: Springer Singapore; 2021. 10.1007/978-981-16-2790-3.

[48] Verma SK, Satyanarayana A, Singh T. Experience of well-being: a cross-generational study in India. J Fam Stud. 2022;28:37–53. 10.1080/13229400.2019.1674181.

[49] Gangopadhyay J, Samanta T. Family matters. Contrib Indian Sociol. 2017;51:338–60. 10.1177/0069966717720962.

[50] Ungar LR, Mahalingam R. “We’re not speaking any more”: a cross-cultural study of intergenerational cut-offs. J Cross Cult Gerontol. 2003;18:169–83. 10.1023/A:1025160602496. 14617955 10.1023/a:1025160602496

[51] Bawdekar M, Ladusingh L. Intergenerational time and monetary support among urban Indian families. Asian Popul Stud. 2012;8:187–205. 10.1080/17441730.2012.684538.

[52] Bardhan DrA. Nature and impact of family care in the life of elderly people: a study in Dhaka city. International Journal of Research and Innovation in Social Science 2024;VIII:2049–60. 10.47772/IJRISS.2024.801151.

[53] Chandra A. Mending Maya: an analysis of aging and intergenerational connection in Delhi. India Innov Aging. 2017;1:787–787. 10.1093/geroni/igx004.2851.

[54] Feng Z, Falkingham J, Liu X, Vlachantoni A. Changes in living arrangements and mortality among older people in China. SSM Popul Health. 2017;3:9–19. 10.1016/j.ssmph.2016.11.009. 29349200 10.1016/j.ssmph.2016.11.009 PMC5768996

[55] Somaiah BC, Yeoh BSA. Grandparenting left-behind children in Javanese migrant-sending villages: trigenerational care circuits and the negotiation of care. Geoforum. 2023;143:103767. 10.1016/j.geoforum.2023.103767. 37456574 10.1016/j.geoforum.2023.103767 PMC10347432

[56] Chen Y, Feeley TH. Social support, social strain, loneliness, and well-being among older adults. J Soc Pers Relat. 2014;31:141–61. 10.1177/0265407513488728.

[57] Baeriswyl M, Girardin M, Oris M. Financial support by older adults to family members: a configurational perspective. J Demogr Economics. 2022;88:167–94. 10.1017/dem.2021.21.

[58] Szydlik M. Sharing lives: adult children and parents. 1st ed. London: Routledge; 2016. 10.4324/9781315647319.

[59] Shariff A. Ethnic identity and parenting stress in South Asian families: implications for culturally sensitive counselling. Can J Couns Psychother. 2009;43:35–46. https://cjc-rcc.ucalgary.ca/article/view/58908.

[60] Choi EY, Ko SH, Jang Y. “Better be dead than grow older:” a qualitative study on subjective aging among older Koreans. J Aging Stud. 2021;59:100974. 10.1016/j.jaging.2021.100974. 34794719 10.1016/j.jaging.2021.100974

[61] Seo BK, Kim JH. Intergenerational coresidence and life satisfaction in old age: the moderating role of homeownership. Appl Res Qual Life. 2022;17:3199–216. 10.1007/s11482-022-10062-y.

[62] Speck S, Müller-Böker U. Population ageing and family change: older people’s perceptions of current changes in family composition in rural Nepal. European Bulletin of Himalayan Research 2020:7–37. 10.4000/ebhr.234.

[63] Woo J. The myth of filial piety as a pillar for care of older adults among Chinese populations. Adv Geriatr Med Res 2020;2. 10.20900/agmr20200012.

[64] Rajan SI, Kumar S. Living arrangements among Indian elderly: new evidence from National Family Health Survey. Econ Polit Wkly. 2003;38:75–80.

[65] Muhammad T, Balachandran A, Srivastava S. Socio-economic and health determinants of preference for separate living among older adults: a cross-sectional study in India. PLoS ONE. 2021;16:e0249828. 10.1371/journal.pone.0249828. 33852617 10.1371/journal.pone.0249828 PMC8046240

[66] Saha AK, Dey SK. Young generation and filial responsibility: an empirical study. Indian Journal of Gerontology. 2013;27:598–609.

[67] Krsteska R, Pejoska-Gerazova V. Family relationships as a risk factor for late life depression. Prilozi. 2010;31:223–35. 21258290

[68] Golden J, Conroy RM, Bruce I, Denihan A, Greene E, Kirby M, et al. Loneliness, social support networks, mood and wellbeing in community-dwelling elderly. Int J Geriatr Psychiatry. 2009;24:694–700. 10.1002/gps.2181. 19274642 10.1002/gps.2181

[69] Zhou G, Wang Y, Yu X. Direct and indirect effects of family functioning on loneliness of elderly Chinese individuals. Curr Psychol. 2018;37:295–301. 10.1007/s12144-016-9512-5.

[70] Falkingham J, Qin M, Vlachantoni A, Evandrou M. Children’s migration and lifestyle-related chronic disease among older parents ‘left behind’ in India. SSM Popul Health. 2017;3:352–7. 10.1016/j.ssmph.2017.03.008. 29349228 10.1016/j.ssmph.2017.03.008 PMC5769047

[71] United Nations. Second World Assembly on Ageing, 8–12 April 2002, Madrid. United Nations 2002. https://www.un.org/en/conferences/ageing/madrid2002. Accessed 22 Mar 2025.

[72] Antman FM. Adult child migration and the health of elderly parents left behind in Mexico. American Economic Review. 2010;100:205–8. 10.1257/aer.100.2.205. 25125699 10.1257/aer.100.2.205 PMC4127891

[73] Marchetti-Mercer MC. Those easily forgotten: the impact of emigration on those left behind. Fam Process. 2012;51:376–90. 10.1111/j.1545-5300.2012.01407.x. 22984975 10.1111/j.1545-5300.2012.01407.x

[74] Scheffel J, Zhang Y. To what extent does rural migration affect the elderly “left-behind.” A paper presented at the 4th SOLE/EALE World Meetings, Montreal, Montreal: 2015.

[75] Xie J, Ding S, Zhong Z, Yi Q, Zeng S, Hu J, et al. Mental health is the most important factor influencing quality of life in elderly left behind when families migrate out of rural China. Rev Lat Am Enfermagem. 2014;22:364–70. 10.1590/0104-1169.3400.2425. 25029045 10.1590/0104-1169.3400.2425 PMC4292632

[76] Adhikari R, Jampaklay A, Chamratrithirong A. Impact of children’s migration on health and health care-seeking behavior of elderly left behind. BMC Public Health. 2011;11:143. 10.1186/1471-2458-11-143. 21366920 10.1186/1471-2458-11-143 PMC3056748

[77] Abas MA, Punpuing S, Jirapramukpitak T, Guest P, Tangchonlatip K, Leese M, et al. Rural–urban migration and depression in ageing family members left behind. Br J Psychiatry. 2009;195:54–60. 10.1192/bjp.bp.108.056143. 19567897 10.1192/bjp.bp.108.056143 PMC2802522

