# Supplementary material for: Understanding intergenerational dynamics and social support’s impact on health and well-being of older adults in South Asia: a scoping review
Source: Syst Rev. 2025 Apr 11;14:86. doi: 10.1186/s13643-025-02833-z (PMC11987332; doi:10.1186/s13643-025-02833-z)
Supplement: Supplementary file 1 — Additional file 1: Search strategy. [file 13643_2025_2833_MOESM1_ESM.docx]

**Additional file 1: Search Strategy**

**PubMed**

(("Older Adults"[Text Word] OR "Aged"[MeSH Terms] OR "Aged"[Text Word] OR "Aging"[Text Word] OR "Elderly"[Text Word] OR "Elders"[Text Word] OR "Senior citizen"[Text Word] OR "Seniors"[Text Word] OR "Older-aged"[Text Word] OR "Older people"[Text Word] OR "Old people"[Text Word] OR "Oldest-Old"[Text Word]) AND (1982/01/01:2023/02/28[Date - Publication] AND "english"[Language]) AND (((("Intergenerational relations"[MeSH Terms] OR "intergenerational relation"[Text Word]) AND "Intergenerational relations"[Text Word]) OR "Intergenerational family relations"[Text Word] OR "Intergenerational care"[Text Word] OR "Intergenerational solidarity"[Text Word] OR "Intergenerational Social Support"[Text Word] OR "Intergenerational Support"[Text Word] OR "Generation gap"[Text Word] OR "Generation gaps"[Text Word] OR "Reciprocal exchange"[Text Word] OR "Intergenerational exchange"[Text Word] OR "interpersonal relationships"[Text Word]) AND (1982/01/01:2023/02/28[Date - Publication] AND "english"[Language])) AND (("asia, southern"[MeSH Terms] OR "South Asia"[Text Word] OR "Southern Asia"[Text Word] OR "Indian subcontinent"[Text Word] OR "India"[MeSH Terms] OR "India"[Text Word] OR "Afghanistan"[MeSH Terms] OR "Afghanistan"[Text Word] OR "Pakistan"[MeSH Terms] OR "Pakistan"[Text Word] OR "sri lanka"[MeSH Terms] OR "sri lanka"[Text Word] OR "Nepal"[MeSH Terms] OR "Nepal"[Text Word] OR "Bhutan"[MeSH Terms] OR "Bhutan"[Text Word] OR "Maldives"[MeSH Terms] OR "Maldives"[Text Word]) AND (1982/01/01:2023/02/28[Date - Publication] AND "english"[Language]))) AND ((1982/1/1:2023/2/28[pdat]) AND (english[Filter]))
